# Cysteamine treatment restores the *in vitro* ability to differentiate along the osteoblastic lineage of mesenchymal stromal cells isolated from bone marrow of a cystinotic patient

**DOI:** 10.1186/s12967-015-0494-0

**Published:** 2015-05-07

**Authors:** Antonella Conforti, Anna Taranta, Simone Biagini, Nadia Starc, Angela Pitisci, Francesco Bellomo, Valentina Cirillo, Franco Locatelli, Maria Ester Bernardo, Francesco Emma

**Affiliations:** Department of Pediatric Hematology/Oncology, IRCCS Bambino Gesù Children’s Hospital, P.le S. Onofrio, 00165 Rome, Italy; Department of Nephrology and Urology, IRCCS Bambino Gesù Children’s Hospital, P.le S. Onofrio, 00165 Rome, Italy; University of Rome, Tor Vergata, Rome; University of Pavia, Pavia, Italy

**Keywords:** Mesenchymal stromal cells, Cystinosis, Osteogenic differentiation, Cysteamine

## Abstract

**Background:**

Cystinosis is a rare autosomal recessive disease caused by mutations of the CTNS gene, which encodes for a lysosomal cystine/H^+^ symporter. In mice, inactivation of the CTNS gene causes intralysosomal cystine accumulation and progressive organ damage that can be reversed, at least in part, by infusion of mesenchymal stromal cells (MSCs). Little is known on the mesenchymal compartment of cystinotic patients. The aim of the study was to test the phenotypical and functional properties of cystinotic MSCs (Cys-MSCs) isolated from bone marrow (BM) aspirate of a patient with nephropathic cystinosis.

**Methods:**

Morphology, proliferative capacity (measured as population doublings), immunophenotype (by flow-cytometry) and immunomodulatory properties (as phytohemagglutinin-induced peripheral blood mononuclear cell proliferation) were analyzed. The osteogenic differentiation potential of Cys-MSCs was evaluated by histological staining (alkaline phosphatase activity, Alzarin Red and von Kossa staining) spectrophotometry and Quantitative Reverse Transcriptase Polymerase Chain Reaction for osteigenic markers in the presence and in the absence of cysteamine. Cys-MSCs were compared with those isolated and expanded *ex vivo* from three healthy donors (HD-MSCs).

**Results:**

Despite a slightly lower proliferative capacity, Cys-MSCs displayed a characteristic spindle-shaped morphology and similar immunephenotype as HD-MSCs. Cys-MSCs and HD-MSCs prevented proliferation of PHA-stimulated allogeneic peripheral blood mononuclear cells to the same extent. After *in vitro* induction into osteoblasts, Cys-MSCs showed reduced alkaline phosphatase (ALP) activity, calcium depositions and expression of ALP and collagen type 1. When Cys-MSCs were treated *in vitro* with increasing doses of cysteamine (50-100-200 μM/L) during the differentiation assay, recovery of Cys-MSCs differentiation capacity into osteoblasts was observed. No difference in adipogenic differentiation was found between Cys-MSCs and HD-MSCs.

**Conclusions:**

Our results indicate that, as compared to HD-MSCs, Cys-MSCs show reduced ability to differentiate into osteoblasts, which can be reverted after cysteamine treatment.

## Background

Cystinosis is an autosomal recessive lysosomal disorder with a reported incidence of 0.5-1.0 per 100,000 live births [1,2]. It is caused by mutations in the *CTNS* gene (17p13.2), which encodes a lysosomal cysteine-proton symporter, termed cystinosin [1-3]. With loss of cystinosin, cystine accumulates and crystallizes within lysosomes, disturbing normal cell biology. Approximately 95% of patients have severe bi-allelic mutations/deletions of CTNS, causing the most severe form of the disease, termed “nephropathic cystinosis” (OMIM 219800). These subjects develop renal Fanconi syndrome during infancy and progress to end-stage renal failure during childhood, if not treated with cysteamine.

Cystinosis is currently treated with cysteamine, a sulfhydryl compound that reduces accumulation of cystine in lysosomes [1]. This drug delays dialysis and kidney transplantation by approximately 5–10 years, but has no significant effect on renal Fanconi syndrome and cannot prevent progression to end-stage renal failure [1,2]. It also delays most of other disease-related long-term complications, but it is not yet clear if it completely prevents them [4,5].

Notably, most children with cystinosis experience growth impairment, even when rickets is adequately treated and when correcting for the degree of renal failure [6]. This raises the hypothesis that bone disease in cystinosis is secondary, at least in part, to the underlying genetic defect, a concept supported by experimental data showing that invalidation of the *CTNS* gene in certain strains of mice is associated neither with renal phosphate wasting nor with renal failure, but causes severe osteopenia and growth retardation [7].

Mesenchymal stromal cells (MSCs) are adult, fibroblast-like multipotent cells, characterized by their ability to differentiate into tissues of mesodermal origin, such as osteoblasts, adipocytes or chondroblasts [8]. These cells are increasingly employed in human medicine, because of their broad immuneregulatory and anti-inflammatory properties [9-12].

The use of MSCs has been reported in an experimental murine model of cystinosis; however, MSCs have not yet been characterized in patients with this disease. Herein, we report the biological and functional characterization of MSCs isolated from a 16-year-old patient with cystinosis who underwent orthopedic surgery for tibial re-alignment [13].

## Methods

### Isolation and culture of BM-derived Cys- and HD-MSCs

Isolation and *ex-vivo* expansion of MSCs from 5 ml of bone marrow (BM) aspirate was performed as previously described [14]. Cells were obtained from a patient harboring a homozygous c.18_21 del GACT *CTNS* mutation (Cys-MSCs) and from 3 healthy donors (HDs), aged 8, 12 and 19 years (HD-MSCs). The Institutional Review Board of Bambino Gesù Children Hospital approved the study design; patients and donors (HDs) gave their written informed consent and the study was conducted according to the Declaration of Helsinki.

Briefly, mononuclear cells (MNCs) were isolated from BM aspirates of the cystinotic patient and from HDs by density gradient centrifugation (Ficoll 1.077 g/ml; Lympholyte, Cedarlane Laboratories Ltd., The Netherlands) and plated in non-coated 75 cm^2^ tissue culture flasks (BD Falcon, NJ, USA) at a density of 160,000/cm^2^ in complete culture medium: DMEM (Gibco, Life Technologies Ltd, Paisley, UK) supplemented with 10% fetal bovine serum (FBS; Euroclone, Milan, Italy), penicillin 50 U/ml, 50 mg/ml streptomycin (Euroclone). Cultures were maintained at 37°C in a humidified atmosphere, containing 5% CO_2_. After 48-hour adhesion, non-adherent cells were removed and culture proceeded with culture medium being replaced twice a week. MSCs were harvested, after reaching ≥80% confluence, using Trypsin (Lonza, Milan, Italy), and were propagated at 4,000 cells/cm^2^.

Cell growth was assessed by cell counting. Population doublings (PDs) were calculated using the formula log_10_(N)/log_10_(2), where N indicates the ratio between the number of cells harvested and number of cell seeded; results are expressed as cumulative PDs from passage 1 to 5 (P1 to P5).

### Characterization of ex-vivo expanded Cys- and HD-MSCs

MSC samples were phenotypically characterized by flow cytometry at P2-P3, using fluorescein isothiocyanate (FITC)- or phycoerythrin (PE)-conjugated monoclonal antibodies specific to CD13, CD14, CD34, CD45, CD73, CD80, CD90, class I-HLA and HLA-DR, CD73, CD105 (BD PharMingen, San Diego, CA). Analyses were performed by immunofluorescence with a FACSCanto flow-cytometer (BD PharMingen); calculations were performed with the FACSDiva software (Tree Star, Inc. Ashland, OR).

Peripheral blood mononuclear cells (PBMCs) were purified by conventional Ficoll separation from heparinized samples obtained from HDs. All samples were processed and used immediately. PBMC proliferation in the presence or in the absence of MSCs was evaluated after stimulation with phytohemagglutinin (PHA-P; Sigma-Aldrich, St Louis, MO) in flat-bottom 96-well tissue culture plates (BD Falcon) containing RPMI 1640 medium (Gibco, Life Technologies Ltd, Carlsbad, CA) supplemented with 10% FBS. Briefly, MSCs were seeded and allowed to adhere overnight before adding 10^5^ PBMCs per well at final MSC:PBMC ratios of 1:2 or 1:10, with or without PHA (4 μg/ml). After 3 days, co-cultures were pulsed with ^3^H-thymidine (1 μCi/well, specific activity 6.7 Ci/mmole, Perkin Elmer, Waltham, MA) and cells were harvested 18 hours later. ^3^H-thymidine incorporation was measured with a Microbeta Trilux 1450 instrument (Perkin Elmer). Results are expressed as mean percentages of PBMC proliferation. All experiments were performed in triplicates in an allogeneic setting (i.e. Cys-MSCs/HD-PBMCs).

To test MSC response to apoptotic stimuli, Cys-MSCs or HD-MSCs were seeded in 24-well culture plates at a density of 5 x 10^4^ MSCs per well and incubated with TNFα (0.03 μg/ml) and actinomycin D (2.5 μg/ml) for 16 hours. Cells were washed with PBS, detached with trypsin and incubated with Annexin V and propidium iodide following manufacturer’s instructions (Annexin V-FITC apoptosis detection kit; Calbiochem, Darmstadt, GE) Early and late apoptotic cells were detected by FACS analysis using FACSCanto flow-cytometer (BD PharMingen) and expressed as percentage of the total cell population.

### Differentiation capacity of ex-vivo expanded Cys- and HD-MSCs

Osteogenic and adipogenic differentiation capacities *in vitro* were assessed at P2-P3 [15]. The osteogenic differentiation capacity was evaluated by incubating cells with αMEM (Euroclone), 10% FBS, penicillin 50 U/ml, 50 mg/ml streptomycin, and 2 mM L-glutamine supplemented with 10^−7^M dexamethasone, 50 mg/ml L-ascorbic acid; starting from day +7 of the culture, 5 mM ß-glycerol phosphate (Sigma-Aldrich, St Louis, MO) was added to the medium. Adipogenic differentiation was evaluated by incubating cells with αMEM, 10% FBS, penicillin 50 U/ml, 50 mg/ml streptomycin, and 2 mM L-glutamine supplemented with 10^−7^M dexamethasone, 50 mg/ml L-ascorbic acid, 100 mg/ml insulin, 50 mM isobutyl methylxanthine, 0,5 mM indomethacin (Sigma-Aldrich) and 5 mM b-glycerol phosphate. After 3 weeks of culture, cells were washed with PBS, fixed with 10% formalin in PBS for 10 minutes and analyzed for adipogenic and osteogenic differentiation and DNA content.

In order to detect osteogenic differentiation, alkaline phosphatase (ALP) activity was assessed by Fast Blue staining (Sigma-Aldrich), whereas the calcium depositions were demonstrated using Alizarin Red staining (Sigma-Aldrich) and von Kossa staining kit according to manufacturer’s procedures (Bio-Optica, Milan, Italy). To detect calcium depositions, MSCs were washed with PBS and incubated with 2% Alzarin Red solution adjusted to pH 5.5 with 0.5% NH_4_OH for 2–5 minutes. Cells were washed with distilled water. Three hundred microliters of 10% cetylpyridimium chloride in phosphate buffer (8 mM Na_2_HPO_4_ + 1.5 mM KH_2_PO_4_) were added and incubated overnight. Alzarin Red release was measured spectrophotometrically at 550 nm and compared to a standard titration curve. Calcium values were related to DNA content from each well. Osteogenic differentiation was also evaluated in the presence of increasing concentrations of cysteamine (50-100-200 μMoles/L), added from day 7 of the differentiation assay. Adipogenic differentiation was evaluated through the morphological appearance of fat droplets that stained positively with Oil Red O (Sigma-Aldrich) [15].

### Total RNA isolation and quantitative reverse transcriptase polymerase chain reaction (qRT-PCR)

After osteogenic differentiation, cells were lysed in Trizol Reagent (Life Technologies) for the isolation of total RNA, in accordance with manufacturer’s instruction. mRNA was reverse transcribed into complementary DNA (cDNA) by a first strand cDNA synthesis kit (Roche Diagnostics, Manhein, Germany). qPCR reactions was performed using KAPA SYBR FAST master mix (KAPABIOSYSTEMS, Boston, MA) and primers for the amplification of COL1A2 (collagen type 1, alpha2), ALP and GAPDH (glyceraldehyde-3-phosphate dehydrogenase) transcripts were employed. qPCR was carried out in duplicate for each data point. The relative quantification of the gene expression was determined normalizing the data of the gene to GAPDH housekeeping gene and using the 2 ^–ΔΔCT^ method.

### Statistical analysis

Statistical analyses were performed using the GraphPad Prism software package. ANOVA was used to compare the percentages of PHA-induced PBMC proliferation; osteogenic differentiation between Cys-MSCs and HD-MSCs was compared by the student *t*-test; the non parametric Mann Whitney test was used for comparisons of the apoptotis assay. A p value lower than 0.05 was considered to be statistically significant.

## Results and discussion

**Figure 1:**
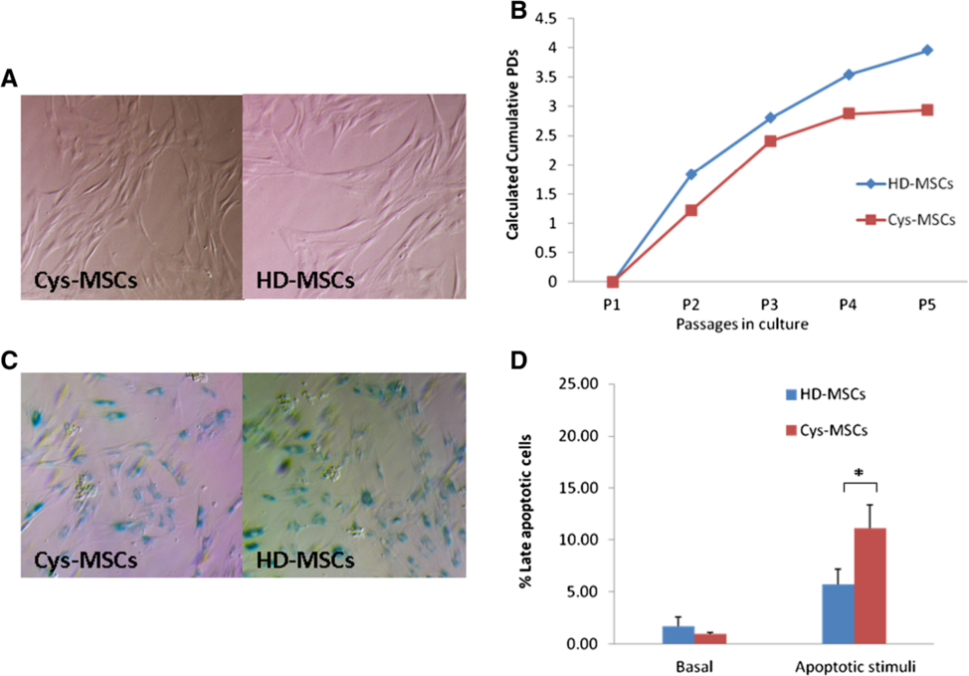
Biological characterization of HD- and Cys-MSCs. **A**: Morphology of bone marrow (BM)-derived mesenchymal stromal cells (MSCs) isolated from the cystinotic patient (Cys-MSCs, on the left) and from one representative healthy donor (HD-MSCs, on the right) at passage 2. Magnification x10. Cys-MSCs display the typical spindle-shape morphology of HD-MSCs. **B**: Calculated population doublings (PDs) from P1 to P5 of HD-MSCs (mean of 3 different HDs) and Cys-MSCs. Cys-MSCs show a lower proliferative capacity, as compared with HD-MSCs. **C**: ß-Galactosidase staining of MSCs isolated from the cystinotic patient (Cys-MSCs on the left) and from one representative healthy donor (HD-MSCs on the right). Senescence of the cells is indicated by the positivity for the staining. **D**: After cysteamine treatment, the percentage of Cys-MSCs late apoptotic cells becomes significantly higher than HD-MSCs’ one.*Indicates a p value lower than 0.05.

**Figure 2:**
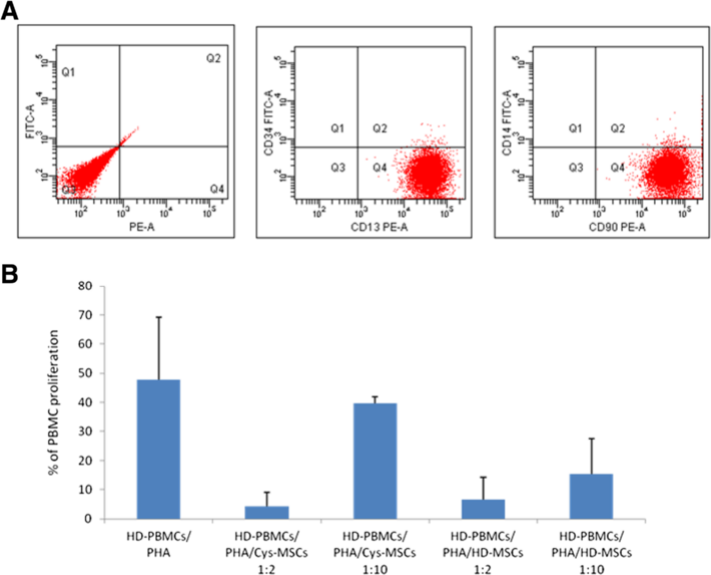
In vitro immunoregulatory properties of HD- and Cys-MSCs. **A**: Immunophenotype of culture-expanded Cys-MSCs. MSCs are positive for CD90 and CD13 surface antigens and negative for CD34 and CD14 molecules. **B**: *In vitro* immunomodulatory effect of HD-MSCs and Cys-MSCs on peripheral blood mononuclear cells (PBMCs) in an allogeneic setting. The graph shows the mean percentage of proliferation of PBMCs stimulated with phytohemagglutinin (PHA) in the absence or in the presence of HD-MSCs or Cys-MSCs at different MSC:PBMC ratios (1:2 and 1:20), calculated by measuring ^3^H-thymidine incorporation after 3-day co-culture. All experiments were performed in triplicates in an allogeneic setting (i.e. Cys-MSCs/HD-PBMCs).

Cys-MSCs were successfully isolated from the BM of a patient with nephropathic cystinosis and were propagated *in vitro*; their biological and functional properties were compared with those of MSCs isolated from BM obtained from three HDs. Cys-MSCs displayed the characteristic spindle-shaped morphology of HD-MSCs (Figure 1A); however, they displayed a lower proliferative capacity, as assessed by PDs (Figure 1B). Cumulative PDs from P1 to P5 were 9.43 and 12.13 (mean; range: 10.45-13.80) for Cys-MSCs and HD-MSCs, respectively. All MSCs were grown in culture until they reached a senescence phase. While Cys-MSCs entered senescence at P13, HD-MSCs reached senescence at P13, P15 and P18, respectively. Senescent state was confirmed in both cell types by beta-Galactosidase staining (Figure 1C). Cys-MSCs showed the same immunophenotype as HD-MSCs; in particular, more than 95% of Cys-MSCs were positive for CD13, CD73, CD90 and CD105, whereas they did not express hematopoietic markers (CD34, CD45, CD14) (Figure 2A)

The immuneregulatory capacity of MSCs was evaluated by measuring their effect on PHA-induced proliferation of allogeneic HD-PBMCs. HD-MSCs exerted a strong inhibitory effect *in vitro* on PHA-induced PBMC proliferation, as previously reported [16]. The mean proliferation rate was decreased from 47.9 ± 21.3% to 6.5 ± 7.8% and 15.3 ± 12.3% in the presence of HD-MSCs at a MSC:PBMC ratio of 1:2 and 1:10, respectively (p < 0.001). Cys-MSCs inhibited proliferation of allogeneic PBMCs to a similar extent (P = NS). Specifically, the proliferation rate was 4.3 ± 4.9% (P = 0.13 as compared with HD-MSCs) and 39.7 ± 2.2% (P = 0.07 as compared with HD-MSCs) in the presence of Cys-MSCs seeded at the same concentrations as above (Figure 2B).

To test if Cys-MSCs displayed a cystinotic phenotype, cystine levels and apoptosis were measured. As expected, Cys-osteoblasts expressed mutated *CTNS* mRNA, reflecting the ubiquitous nature of cystinosin (data not shown). Cys-osteoblasts demonstrated increased cystine concentrations (17.85 ± 1.76 nmol/mg proteins in Cys-cells versus 0.37 ± 0.05 nmol/mg proteins in HD-cells), which decreased substantially after treatment with cysteamine (5.7 ± 0.006 nmol/mg protein). The basal apoptotic rate of Cys-MSCs was similar to that of HD-MSCs; however, as expected, late apoptotic cells were significantly more abundant after stimulation in Cys-MSCs cultures (11.1 ± 2.2% in Cys-MSCs and 5.7 ± 1.5 in HD-MSCs; p = 0.02) (Figure 1D). No difference was observed in early apoptosis between Cys-MCSs (28.6 ± 3.2%) and HD-MSCs (31.2 ± 3.0%).

**Figure 3:**
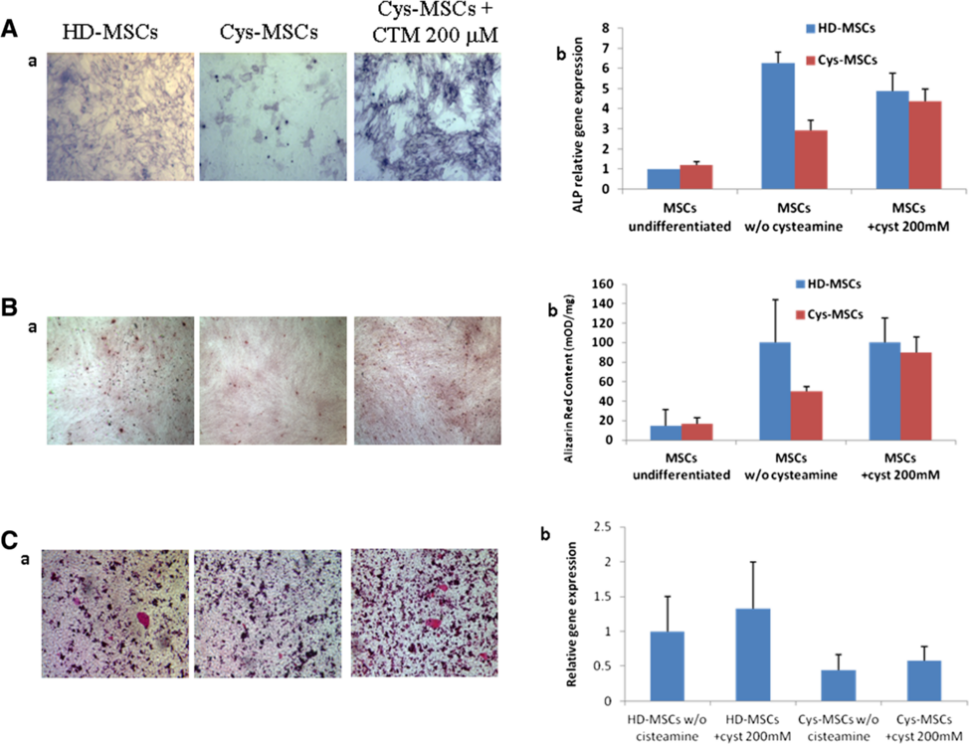
Osteogenic differentiation capacity of HD- and Cys-MSCs. **A: a)** alkaline phosphatase (ALP) staining of Cys-MSCs as compared with HD-MSCs, in the absence and in the presence of cysteamine (CTM 200 μM). **A: b)** Quantitative Reverse Transcriptase Polymerase Chain Reaction (qRT-PCR) for ALP in HD- and Cys-MSCs in the absence and in the presence of CTM. **B: a)** Alzarine Red staining of Cys-MSCs as compared with HD-MSCs, in the absence and in the presence of CTM. **B: b)** Quantification of Alzarin Red staining in HD- and Cys-MSCs by spectrophotometry. The amount of Alzarin Red is related to the amount of DNA extracted from the same wells and is expressed in mOD/min/μg. Each bar represents the mean +/−SD of three experiments. **C: a)** Von Kossa staining of Cys-MSCs as compared with HD-MSCs, in the absence and in the presence of CTM. **C:, b)** qRT-PCR for collagen type 1 in HD- and Cys-MSCs in the absence and in the presence of CTM. The relative quantification of the gene expression was determined normalizing the data of the gene to GAPDH housekeeping gene and using the 2 ^–ΔΔCT^ method.

The differentiation ability into osteoblasts of Cys-MSCs was analyzed *in vitro* by evaluating alkaline phosphatase activity, mineralization and osteogenic marker gene expression levels (Figure 3). After incubation with osteo-induction medium, Cys-MSCs demonstrated limited ALP activity as compared with HD-MSCs (Figure 3A, panel a). Cells were, then, treated with cysteamine to investigate if treatment could restore their osteogenic differentiation capacity. When Cys-MSCs were incubated with increasing concentrations of cysteamine, ALP activity was completely restored (Figure 3A panel a). While ALP gene expression levels were similar in undifferentiated HD- and Cys-MSCs; after osteogenic stimulation, they were 6.27 ± 0.5 and 2.91 ± 0.5 in HD- and Cys-MSCs, respectively (p < 0.005). A significant increase in ALP gene expression levels was measured in Cys-MSCs after cysteamine treatment (4.37 ± 0.6; p = 0.035 as compared with untreated Cys-MSCs), suggesting a beneficial effect of the drug on ALP activity and expression (Figure 3A, panel b). The mineralization ability was also analyzed by Alzarin Red and von Kossa staining. While HD-MSCs were able to mineralize, Cys-MSCs showed little calcium depositions after osteogenic stimulation (Figure 3B, panel a for Alzarin Red and Figure 3C, panel a for von Kossa). When cells were treated with cysteamine at concentrations ≥100 μmol/L, the ability of Cys-MSCs to form *in vitro* calcium depositions positive for Alzarin Red was restored (p = 0.005, as compared with non-treated cells; Figure 3B panel b). Finally, gene expression levels of the osteogenic marker collagen type 1, alpha 2 were analyzed. A trend for an increased level of COL1A2 in Cys-MSCs was observed when cysteamine was added to the osteogenic medium (p = NS) (Figure 3C, panel b).

No differences in adipogenic differentiation capacity was observed between HD-MSCs and Cys-MSCs; cysteamine did not modify cell behavior (data not shown).

Despite very significant progress in its treatment, nephropathic cystinosis remains a severe condition, markedly impairing the patient quality of life. Mechanisms causing cell dysfunction in this disease have not been fully elucidated and only limited studies have investigated bone disease. Renal failure, phosphate wasting and hypothyroidism may in part explain growth retardation and osteopenia observed in these patients, but clinical and experimental observations also suggest that cystinosis-specific factors play an important role. A simple way of assessing if abnormalities are secondary to cystine accumulation is to treat animals or cell models with cysteamine. In this respect, clinical data suggest that bone abnormalities *in vitro* should be ameliorated by cystine depletion. Indeed, it is becoming increasingly rare to observe very severely growth retarded cystinotic children in the era of cysteamine treatment. Assessing the clinical impact of cysteamine and its mode of action on bone structures in these patients can be difficult, due to frequent coexistence of confounding factors, such as growth hormone therapy [17]. Moreover, cysteamine can also exert indirectly its effect on statural growth, by extending the number of years with normal renal function and preventing hypothyroidism. Finally, early treatment of Fanconi syndrome with phosphate supplementation and with vitamin D impacts positively on bone mineralization, which may mask the effects of cysteamine.

However, several clinical studies have now provided convincing indications that cysteamine may improve growth and, *per se*, may ameliorate bone disease [5,18,19]. Our results support this concept. First, they demonstrate that Cys-MSCs differentiate poorly into osteoblasts. This is consistent with altered bone and poor growth of *CTNS* −/− mice, even in the absence of phosphate wasting and of renal failure [7]. Secondly, our results show that, *in vitro*, differentiation into osteoblasts can be restored by treating cells with cysteamine, this finding being consistent with the clinical data mentioned above. Despite the fact that our data concern a single patient, the observed *in vitro* response to cysteamine favors the hypothesis of a specific defect present in cystinotic cells.

The mechanisms by which cysteamine allows normal osteoblast differentiation of Cys-MSCs are currently unknown. Notably, we recently reported that intracellular cystine crystals are potent activators of the inflammosome [20]. Hypothetically, bone disease in cystinosis could be secondary to the abnormal production of inflammatory cytokines, as observed in other chronic inflammatory diseases. However, cystine crystals do not form *in vitro.* Instead, normal differentiation of Cys-MSCs into adipocyte suggests inhibition of an osteoblast-specific differentiation program by lysosomal cystine. In cystinotic proximal tubular epithelial cells, the ZO-1-associated Y-box factor (ZONAB) translocates to the nucleus and cells undergo a de-differentiation program [21]. This factor is normally located in the cytosol through its binding to tight junctions [22]. The link between lysosomal cystine accumulation and ZONAB release has not been elucidated yet. Similar mechanisms could prevent the formation of mature osteoblasts and may explain the bone phenotype observed in cystinosis. Given the limitations of the observation in only one Cys-MSC sample and the difficulty of obtaining mechanicistic information from a single patient, this *ex vivo* Cys-MSC model could be particularly suitable to test this hypothesis in properly designed future studies.

## Conclusions

Our results indicate that, as compared to HD-MSCs, Cys-MSCs are defective in their ability to differentiate *in vitro* into osteoblasts. This finding is in line, in general, with the growth retardation and osteopenia observed in cystinotic patients and, in particular, with the observation of a reduced number of osteoblasts in the bone biopsy in our patient. The impaired capacity to form bone *in vitro* in Cys-MSCs can be restored after cysteamine treatment; this finding favors the hypothesis of a specific bone defect present in cystinotic cells. This *ex vivo* Cys-MSC model could be particularly suitable to investigate bone defects in Cystinosis.

## References

[1] Gahl WA, Thoene JG, Schneider JA (2002). Cystinosis. N Engl J Med.

[2] Levtchenko E, van den Heuvel L, Emma F, Antignac C (2014). Clinical utility gene card for: cystinosis. Eur J Hum Genet.

[3] Wilmer MJ, Emma F, Levtchenko EN (2010). The pathogenesis of cystinosis: mechanisms beyond cystine accumulation. Am J Physiol Renal Physiol.

[4] Brodin-Sartorius A, Tête MJ, Niaudet P (2012). Cysteamine therapy delays the progression of nephropathic cystinosis in late adolescents and adults. Kidney Int.

[5] Gahl WA, Balog JZ, Kleta R (2007). Nephropathic cystinosis in adults: natural history and effects of oral cysteamine therapy. Ann Intern Med.

[6] Van Stralen KJ, Emma F, Jager KJ (2011). Improvement in the renal prognosis in nephropathic cystinosis. Clin J Am Soc Nephrol.

[7] Cherqui S, Sevin C, Hamard G (2002). Intralysosomal cystine accumulation in mice lacking cystinosin, the protein defective in cystinosis. Mol Cell Biol.

[8] Friedenstein AJ, Deriglasova UF, Kulagina NN (1974). Precursors for fibroblast in different populations of hematopoietic cells as detected by the in vitro colony assay method. Exp Hematol.

[9] Ball LM, Bernardo ME, Roelofs H (2007). Cotransplantation of ex vivo expanded mesenchymal stem cells accelerates lymphocyte recovery and may reduce the risk of graft failure in haploidentical hematopoietic stem-cell transplantation. Blood.

[10] Le Blanc K, Frassoni F, Ball L (2008). Mesenchymal stem cells for treatment of steroid-resistant, severe, acute graft-versus-host disease: a phase II study. Lancet.

[11] Ball LM, Bernardo ME, Roelofs H (2013). Multiple infusions of mesenchymal stromal cells induce sustained remission in children with steroid-refractory, grade III-IV acute graft-versus-host disease. Br J Haematol.

[12] Ciccocioppo R, Bernardo ME, Sgarella A (2011). Autologous bone marrow-derived mesenchymal stromal cells in the treatment of fistulising Crohn's disease. Gut.

[13] Syres K, Harrison F, Tadlock M (2009). Successful treatment of the murine model of cystinosis using bone marrow cell transplantation. Blood.

[14] Bernardo ME, Zaffaroni N, Novara F (2007). Human bone marrow-derived mesenchymal stem cells do not undergo transformation after long-term in vitro culture and do not exhibit telomere maintenance mechanisms. Cancer Res.

[15] Int’Anker PS, Noort WA, Scherjon SA (2003). Mesenchymal stem cells in human second-trimester bone marrow, liver, lung, and spleen exhibit a similar immunophenotype but a heterogeneous multilineage differentiation potential. Haematologica.

[16] Di Nicola M, Carlo-Stella C, Magni M (2002). Human bone marrow stromal cells suppress T-lymphocyte proliferation induced by cellular or nonspecific mitogenic stimuli. Blood.

[17] Wühl E, Haffner D, Offner G (2001). Long-term treatment with growth hormone in short children with nephropathic cystinosis. J Pediatr.

[18] Greco M, Brugnara M, Zaffanello M (2010). Long-term outcome of nephropathic cystinosis: a 20-year single-center experience. Pediatr Nephrol.

[19] Kleta R, Bernardini I, Ueda M (2004). Long-term follow-up of well-treated nephropathic cystinosis patients. J Pediatr.

[20] Prencipe G, Caiello I, Cherqui S (2014). Inflammasome activation by cystine crystals: implications for the pathogenesis of cystinosis. J Am Soc Nephrol.

[21] Raggi C, Luciani A, Nevo N (2014). Dedifferentiation and aberrations of the endolysosomal compartment characterize the early stage of nephropathic cystinosis. Hum Mol Genet.

[22] Balda MS, Garrett MD, Matter K (2003). The ZO-1-associated Y-box factor ZONAB regulates epithelial cell proliferation and cell density. J Cell Biol.

